# A Clinical Study of the Pulse Wave Characteristics at the Three Pulse Diagnosis Positions of Chon, Gwan and Cheok

**DOI:** 10.1093/ecam/nep150

**Published:** 2011-05-02

**Authors:** Young J. Jeon, Jaeuk U. Kim, Hae J. Lee, Jeon Lee, Hyun H. Ryu, Yu J. Lee, Jong Y. Kim

**Affiliations:** ^1^Constitutional Biology and Medical Engineering Research Center, Korea Institute of Oriental Medicine (KIOM), 461-24 Jeonmin-dong, Yuseong-gu, Daejeon 305-811, Republic of Korea; ^2^Department of Oriental Biomedical Engineering, Daegu Hanny University, Yugok-dong, Gyeongsan-si, Gyongsangbuk-do, Republic of Korea

## Abstract

In this work, we analyze the baseline, signal strength, aortic augmentation index (AIx), radial AIx, time to reflection and P_T2 at Chon, Gwan, and Cheok, which are the three pulse diagnosis positions in Oriental medicine. For the pulse measurement, we used the SphygmoCor apparatus, which has been widely used for the evaluation of the arterial stiffness at the aorta. By two-way repeated measures analysis of variance, we tested two independent measurements for repeatability and investigated their mean differences among Chon, Gwan and Cheok. To characterize further the parameters that were shown to be different between each palpation position, we carried out Duncan's test for the multiple comparisons. The baseline and signal strength were statistically different (*P* < .05) among Chon, Gwan and Cheok, respectively, which supports the major hypothesis of Oriental medicine that all of the three palpation positions contain different clinical information. On the other hand, aortic AIx and time to reflection were found to be statistically different between Chon and the others, and radial AIx and P_T2 did not show any difference between pulse positions. In the clinical sense, however, the aortic AIx at each palpation position was found to fall within the 90% confidence interval of normal arterial compliance. The results of the multiple comparisons indicate that the parameters of arterial stiffness were independent of the palpation positions. This work is the first attempt to characterize quantitatively the pulse signals at Chon, Gwan and Cheok with some relevant parameters extracted from the SphygmoCor apparatus.

## 1. Introduction

The pulse wave is a pressure wave along the artery that offers various information on cardiovascular conditions. Its detailed waveform is determined by the sum of the incident wave caused by the ventricular output of the blood and the reflected wave from the peripheral blood vessel [1, 2]. The ratio between the pulse pressure and the augmented pressure is called the augmentation index (AIx), which is widely used to clinically examine the stiffness of the artery, together with the pulse wave velocity [3, 4]. A popular instrument to test the stiffness of the aortic artery by calculating the AIx is the SphygmoCor apparatus (AtCor Medical, Australia). This instrument uses the method of applanation tonometry to first measure the pulse waveform at the radial artery and then synthesize the aortic pulse wave by a transfer function and finally calculate the aortic AIx to evaluate the stiffness of the aortic artery [5, 6]. The SphygmoCor apparatus is known for high reproducibility and repeatability in its measurements, as verified by several clinical works by Wilkinson et al. [7], Seibenhofer et al. [8] and Filipovský et al. [9].

**Figure 1:**
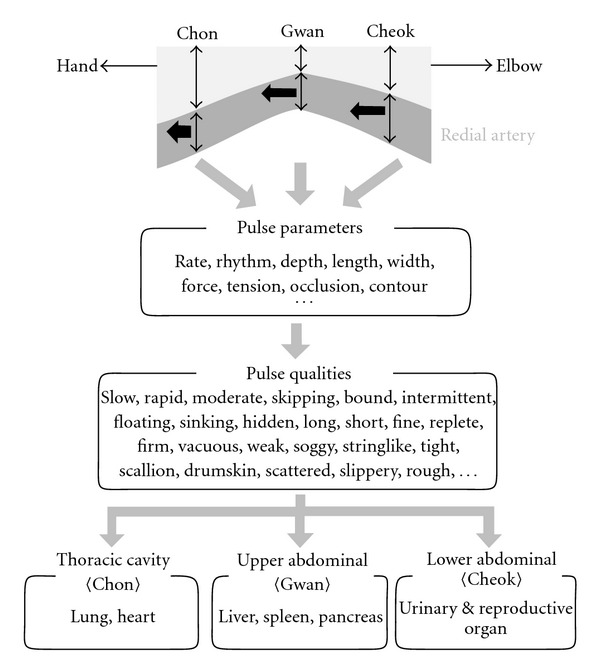
Diagram of commonly accepted hypothetical correspondence between palpation positions and major organ systems.

In Oriental medicine, the pulse diagnosis has been utilized widely as one of the four important diagnosis methods, which are inspection, listening and smelling, inquiring and palpation [10, 11]. For the pulse diagnosis, Oriental medical doctors divide the terminal region of the radial artery into three adjacent intervals called Chon, Gwan and Cheok, and use the three fingers of index, middle and ring fingers simultaneously or individually to determine various characteristic features of the pulse wave [12]. By these features, one can obtain detailed information on various illnesses and their progress on a systematic level as well as the basic health conditions of patients. Each palpation position is believed to reflect the health condition and functioning of different organs. A popular hypothesis of such viewpoint is that the pulse at Chon describes the functioning of organs of the upper region of the trunk and the thoracic cavity such as lung and heart. Similarly, the pulse at Gwan and Cheok is ascribed respectively to the upper abdominal cavity (liver, spleen, and pancreas) and lower abdominal cavity (urinary and reproductive organs) [13–16].  Figure 1 depicts a more detailed hypothetic connection between each palpation position and respective organs.

The traditional method by human fingers relies entirely on the doctor's own experiential judgment, which may be affected by various environmental conditions that include the measurer's own sensibility fluctuation. Therefore, the quantification and standardization of the pulse diagnosis is in urgent need, which requires the transformation of doctors' subjective feelings into objective physical quantities. Indeed, some efforts toward the quantification and standardization are going on. For example, pulse diagnosis systems based on the arrays of piezo-resistive sensors are being developed and updated, clinical data using pulse diagnostic instruments for statistical purposes are being collected and more fundamental studies such as the blood flow dynamics along the radial artery are under investigation [17–19]. However, these efforts are still in a fertilizing stage and reliable instruments for measuring and analyzing the pulse that can replace doctors' palpation by fingers are not yet invented.

In this work, using the SphygmoCor apparatus that is used extensively in aortic stiffness examination, we measured the pulse waves independently at the three palpation positions of Chon et al. and analyzed the characteristic features of the waveform such as the aortic AIx, radial AIx, time to reflection and P_T2, which correspond to the time it takes for the reflected wave to arrive at the position of interest after it bounces at the end of the peripheral artery, and the baseline and the signal strength, which correspond to the hold-down pressure and the pulse pressure, respectively. We will focus on the quantitative differences of these parameters at the three pulse positions of Chon, Gwan and Cheok.

## 2. Methods

### 2.1. Subjects

In the choice of the subjects, we attempted to minimize variations depending on gender, age, and health conditions. For this purpose, we chose 20 healthy males aged in their twenties who had not had any cardiovascular disease, diabetes, or hypertension, and had not had any surgery at the wrist joint and blood vessels. At the same time, the subjects satisfied further requirements such as systolic blood pressure <130 mmHg and diastolic pressure <80 mmHg, and being within the normal range (18.5–24.9 kg/m^2^) of body mass index.  Table 1 lists details of the physiological data of the subjects. The subjects were asked to answer a detailed questionnaire on medication, alcoholic drinking and smoking, and all of them filled up an agreement form on the experiment. Each subject was forbidden from smoking and drinking for 1 and 6 hours, respectively, before the experiment. From the subject selection to the experimental method and data manipulation, the procedure went through the approval of the Institutional Review Board of the Oriental Medicine Hospital at Daejeon.

### 2.2. Pulse Measurement

After the questionnaire was administered, using an auscultatory method blood pressure was measured twice over a 2 minute interval and was averaged. After the subjects had 5 minutes of rest in comfort, we measured the pulse of each subject at the left radial artery in the supine position twice with a 2 minute interval at rest in the sitting position.

**Figure 2:**
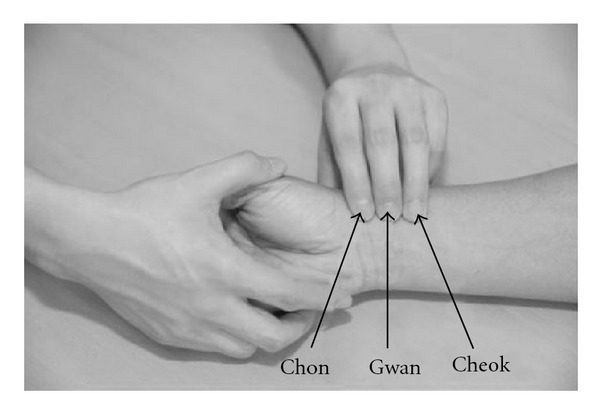
Positioning of Chon, Gwan, and Cheok.

**Figure 3:**
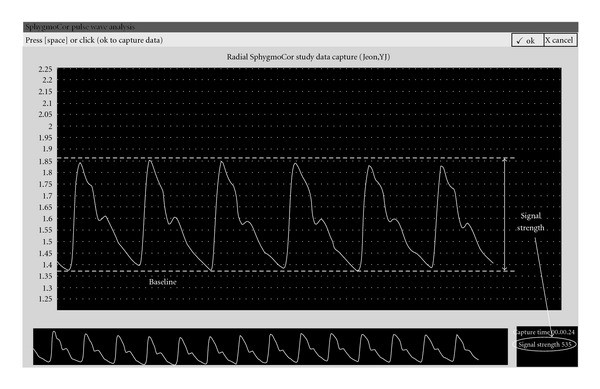
Definition of signal strength and baseline in the pulse waveform.

 The three palpation positions for the pulse diagnosis were within ±15 mm from the prominent bone, as schematically shown in Figure 2 [20]. The accurate positions of Chon, Gwan, and Cheok, were marked by doctors in Oriental medicine. To avoid any bias in data collection, we randomly chose the measuring positions among Chon, Gwan and Cheok for the second measurement of each subject. The measurement was carried out by the SphygmoCor apparatus. The optimal positioning of the sensor and the hold-down pressing is operated manually, which makes the measured pulse sensitive to the movement of the operating hand and requires the data qualification process to guarantee the reliability of the measurement. Following the SphygmoCor manual [21], the signal strength, which represents the difference between the maximum and the minimum of the pulse waveform for 5 s as depicted in Figure 3, was kept above 360 during the measurement. Furthermore, we applied strict criteria to filter out the spoiled data due to the movement of the pressing hand. Firstly, the operator index (OI) should be 90 or above and simultaneously the parameters such as the average pulse height, pulse height variation, diastolic variation, shape deviation and maximum dP/dT should be within the allowed level of tolerance (green-colored range in the manual). Additionally, the signal strength and the baseline of the second measurement should lie within the window of ±100 and ±200, respectively, compared with the first measurement [21]. 

**Figure 4:**
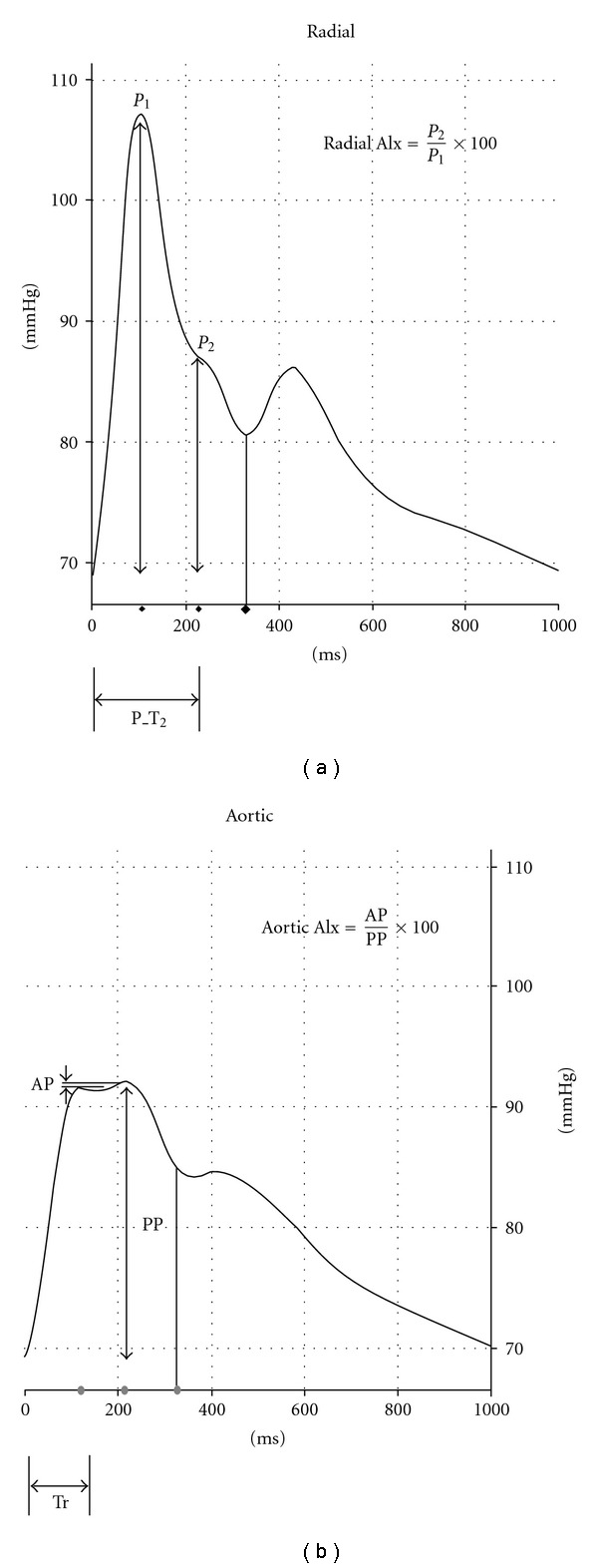
(a) Definition of the radial AIx and P_T2. (b) Definition of the aortic AIx and Tr. (PP: pulse Pressure, AP: augmented Pressure, Tr: time to reflection).

Figure 3 shows an example of the signal strength and baseline captured on the apparatus screen. Besides the signal strength and the baseline, we estimated the aortic AIx, radial AIx, time to reflection and P_T2, which are introduced in Figure 4. The radial AIx is calculated directly from the measured pulse waveform at the radial artery, whereas the aortic AIx is calculated by employing a transfer function to synthesize the aortic pulse waveform using a raw pulse signal at the radial artery. Following the standard procedure, we convert the aortic AIx to the AIx@75, which corresponds to the AIx at the assumed heartbeat rates of 75. The time to reflection indicates the reflected wave's return time to the aorta from the boundary of the peripheral artery. Similarly, P_T2 indicates the return time of the reflected wave to each palpation position.

### 2.3. Statistical Method

For the statistical analysis, we used the SAS 9.1 program. By two-way repeated measures analysis of variance (ANOVA), we examined the repeatability of the baseline, signal strength, aortic AIx, radial AIx, time to reflection and P_T2 of the waveform, for the two independent measurements at each palpation position of Chon, Gwan, and Cheok. We also tested for their mean differences between the palpation positions. For the cases showing significant differences between their measuring positions, we carried out Duncan's test for the multiple comparisons. The significance criterion for all the parameters was set at 5% of the statistical significance level (*P* < .05).

## 3. Results

### 3.1. Repeatability of Measurement

**Figure 5:**
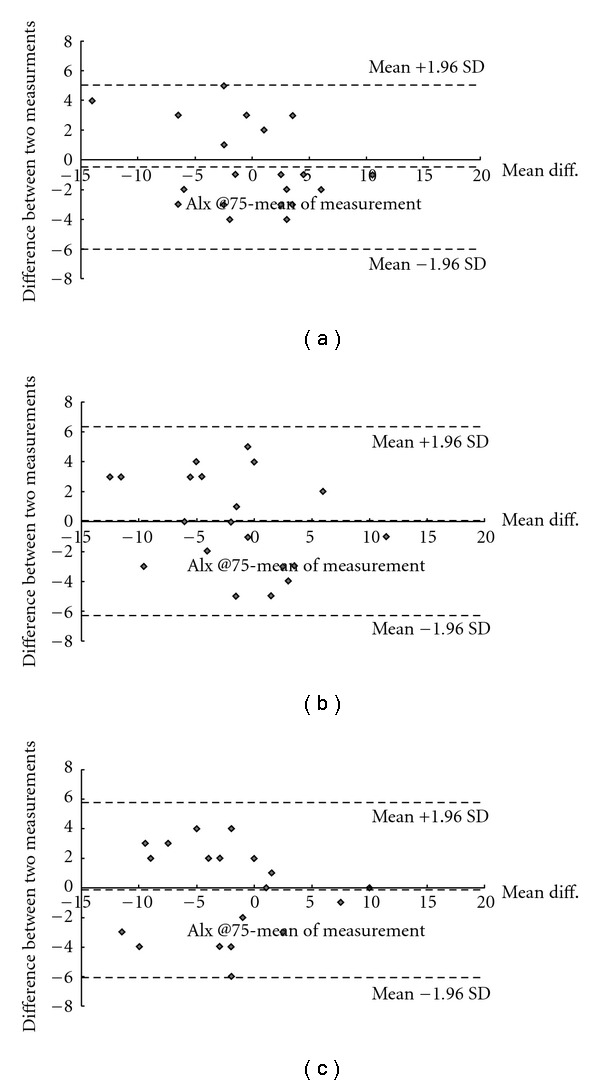
Bland–Atman plots of AIx@75 at Chon, Gwan, and Cheok, within the observer repeatability of AIx@75 (a) at Chon, (b) at Gwan and (c) at Cheok.

To test the repeatability for the two independent sets of pulse, we performed two-way repeated measures ANOVA, and examined the mean differences of the baseline, signal strength, AIx@75, radial AIx, time to reflection, and P_T2 between the two datasets. The results showed no mean difference in any parameter (*P* > .05).  Figure 5 shows the Bland–Altman plot for the repeated measures of the AIx@75 at each pulse position. It indicates that the mean differences between the two independent measures were found to be negligible, for example, we obtained the differences within 1.96 × standard deviation (SD) irrespective of the mean values at each measurement position. For Chon, Gwan, and Cheok, the mean and the standard error of the mean (mean ± SEM) of the differences in the AIx@75 between the two measures were estimated to be –0.45 ± 0.63, 0.05 ± 0.72, and −0.15 ± 0.68, respectively. 

### 3.2. The Mean Differences between Different Measuring Positions

**Figure 6:**
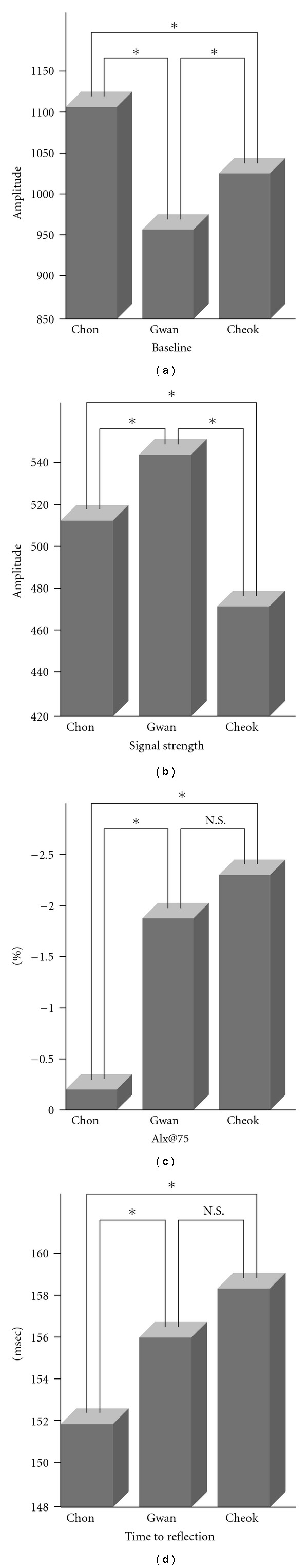
(a)–(d) Bar-plot for the quantities that showed significant differences from the two-way repeated measures ANOVA (for the complete result of the test, refer Table 2). (*P < .05, N.S.: no significance).

By two-way repeated measures ANOVA, for all the parameters except for the radial AIx and P_T2, we observed significant differences (*P* < .05) in their mean values between different measuring positions. The ANOVA results are tabulated in Table 2, in the form of mean ± SD. They show that the mean baseline decreased in the order of Chon, Cheok, and Gwan, while the signal strength decreased in the order of Gwan, Chon and Cheok. The AIx@75 increased, in the negative direction, in the order of Chon, Gwan, and Cheok. Likewise, the time to reflection increased in the order of Chon, Gwan, and Cheok. The quantities that showed statistical differences are also bar-plotted in Figure 6. 

### 3.3. Multiple Comparisons of the Parameters at Different Measuring Positions

Finally, to examine the mean differences of the four parameters yielding statistical differences between the pulse positions, we carried out Duncan's test for the multiple comparisons. The results presented in Tables 3 and 4 show that, in the baseline and signal strength, the mean values were different between each pulse positions of Chon, Gwan, and Cheok (*P* < .05). On the other hand, the AIx@75 and the time to reflection did not differ between Gwan and Cheok, while mean difference was found between Chon and the others.

## 4. Discussion and Conclusions

In this work, using the SphygmoCor apparatus, we studied the characteristic behaviors of the baseline, signal strength, AIx@75, radial AIx, time to reflection and P_T2 at the three popular positions of pulse diagnosis used in Oriental medicine, that is, at Chon, Gwan, and Cheok. For this purpose, for a group of healthy subjects, we measured the pulse signals twice at each pulse diagnosis position and obtained the accurate data over 90 of the operator index. By two-way repeated measures ANOVA, we found that, firstly, the two sets of measurement were repeatable, and secondly, the mean differences of the baseline, signal strength, AIx@75 and the time to reflection were different (*P* < .05) between each palpation position. More specifically, the mean of the baseline decreased in the order of Chon, Cheok, and Gwan, while the signal strength decreased in the order of Chon, Gwan, and Cheok,. The AIx@75 decreased in the order of Chon, Gwan, and Cheok, and the time to reflection increased in the same order (note the minus sign in the AIx@75 in Figure 6). This strong correlation between the AIx@75 and the time to reflection is physically reasonable; the AIx@75 decreases more for the longer time to reflection. In parallel, we performed Duncan's test and found the baseline and the signal strength between each measurement position were statistically distinct, implying that those quantities at different positions were quantitatively different. On the other hand, the AIx@75 was the largest on average at Chon and smallest at Cheok, while it did not differ statistically between Gwan and Cheok. The same characteristic behavior with the AIx@75 was also found in the time to reflection.

Lee et al. [22] studied the characteristics of the radial artery at the proximity of the three pulse positions using ultrasonic waves. In their study, the depth of the radial artery, defined by the distance between the epidermis and the upper wall of the blood vessel was deepened in the order of Gwan, Cheok, and Chon. Noting that to measure the maximal pulse strength the hold-down pressure between the sensor and the blood vessel should be applied until it equilibrates with the inner pressure of the vessel, their result indicates that the optimal hold-down pressure yielding the maximal pulse strength increases in the order of Gwan, Cheok, and Chon. This is in agreement with our finding that the average of the baseline was the largest in Chon and smallest in Gwan.

**Figure 7:**
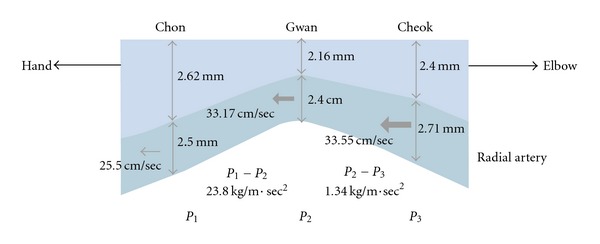
Thickness and depth of the blood vessel and the blood velocity in it at the three palpation positions [22].

Lee et al. [22] calculated the differences in the blood pressure between adjacent palpation positions, for example, positions 1 and 2, by applying the explicitly measured blood velocity *ν* (blood density *ρ* = 1.06 g/cm^3^) at these positions [23], using Bernoulli's flow equation

(1) $$P_{1}-P_{2}=0.5\times \rho (v_{1}^{2}-v_{2}^{2}),$$

where *P*_1_ and *P*_2_ are the pressures at positions 1 and 2, respectively. (1) describes energy conservation, where the sum of the kinetic energy (*∝ρ*v^2^) and the potential energy (*∝P*) are conserved at each position. As shown in Figure 7, the estimated vessel pressure was largest at Chon and the pressure gradient between Chon and Gwan was 23.8 kg m^−1^ s^−2^ which is equivalent to 0.18 mmHg. It implies that the average blood pressure is not noticeably different between the pulse positions. In our experiment, the signal strength at Gwan was measured the largest and the smallest at Cheok. The largest signal strength at Gwan can be understood well. First of all, the depth of the blood vessel from the epidermis is the shortest at Gwan, which causes the signal strength of the vessel to be transmitted to the epidermis without significant loss. In addition, the radial artery beneath Gwan lies just above the prominent bone, which works as a support for the vessel when it is pressed down by a sensor. Note that the optimal hold-down pressure yielding the maximal pulse signal is different from region to region, and therefore the signal strength is not a simple function of the depth of the blood vessel or the pressure in it alone. For this reason, further theoretical and experimental efforts are required to understand why the signal strength at Cheok is smaller than the one at Chon.

Duncan's test shows that the AIx@75 and time to reflection estimated at Chon are statistically distinct from those at Gwan or Cheok (*P* < .05), while the radial AIx and P_T2 at all the measurement positions do not differ, as shown in Table 2. In analogy with the aortic AIx that estimates the stiffness of the aortic artery, the radial AIx is closely related to the stiffness of the radial artery at the measurement points. Since the three measurement points are next to each other, the arterial compliances at these positions are expected not to be different. Therefore, our result showing that the radial AIx and P_T2 do not differ between those adjacent positions (*P* > .05) follows general expectation, and it is in agreement with the property of the string-like pulse in Oriental medicine as well, which appears when the arterial wall is taut and is known to be detected at all palpation positions simultaneously [20]. Despite the estimated waveform of the aortic pulse depending on the specification of the radial-to-aortic transfer function, it is known that the high-frequency component of the estimated aortic wave such as the AIx@75 (and time to reflection) is more sensitive to the detailed behavior of the original wave than is the low-frequency part, that is, the systolic pressure. In addition, the estimation of the aortic AIx via the transfer function for young healthy group is reported to be rather erratic [24]. It can explain the reason for the statistically different AIx@75 (time to reflection) between Chon and the others, in spite of statistically non-distinctive radial AIx (and P_T2). We emphasize, however, that the aortic AIx at different positions all fall inside the 90% confidence interval of the healthy adults in view of the arterial stiffness [21]. Therefore, as far as the clinical significance is concerned, for the diagnosis of the arterial stiffness pinpoint accuracy may not be necessary as long as the palpation is around the prominent bone. Further study on the AIx for older subjects and a group of patients with cardiovascular disease is needed for more confirming results.

In conclusion, we investigated the characteristic behaviors of the baseline, signal strength, aortic AIx, radial AIx, time to reflection and P_T2 independently at Chon, Gwan, and Cheok, which are located adjacent to each other at the proximity of the prominent bone and are considered the three major palpation positions in Oriental medicine. The radial AIx and P_T2 did not show any statistical difference between the palpation positions, in accordance with the property of the string-like pulse. On the other hand, the estimated AIx@75 and the time to reflection were statistically different (*P* < .05) between Chon and Gwan, and between Chon and Cheok, reflecting that the estimated aortic AIx (and time to reflection) is rather sensitive to the detailed structure of the original waveform. However, we note that clinically the aortic AIx estimated from each palpation position falls within the 90% confidence interval of the normal arterial compliance, suggesting that the three palpation positions are equally acceptable to estimate the aortic AIx. A subsequent study on AIx for older and/or hypertensive subjects will help us gain a more conclusive result. On the other hand, the baseline (hold-down pressure) and the signal strength (pulse pressure), which are among the major parameters used in pulse diagnosis in Oriental medicine, were statistically different (*P* < .05) among the three palpation positions. This result supports the major hypothesis of Oriental medicine that all of the three palpation positions contain different clinical information, and further studies to quantify and clarify the meaning of various types of the pulse waveforms are needed for the standardization of Oriental medicine.

To our knowledge, this work is the first attempt to study quantitatively the characteristics of the radial artery at the three pulse diagnosis positions of Chon, Gwan, and Cheok, using the SphygmoCor apparatus, which has been popularly used for clinical purposes, for example, to examine the stiffness of the blood vessel, by a non-invasive measurement of the pulse wave. Our study may motivate further research activities toward quantification of various properties of pulse signals at the three important diagnosis positions, and it may be used as a reference for the development of the next generation apparatus of pulse diagnosis.

## Funding

This work was supported by the Korea Ministry of Knowledge Economy (10028438).

## Figures and Tables

**Table 1:** Subject characteristics.

Characteristic	Value (mean ± SD)
Number	20 (male)
Age (years)	23.35 ± 1.84
HR (bpm)	63.1 ± 6.79
SBP (mmHg)	115.5 ± 8.39
DBP (mmHg)	72.5 ± 7.23
Height (cm)	175.4 ± 4.63
Weight (kg)	69.1 ± 8.29
Body mass index	22.41 ± 1.92

HR, Heart Rate.

**Table 2:** Results of two-way repeated measures ANOVA for mean differences at the three palpation positions.

	Chon		Gwan		Cheok		*P*-value
	First	Second	First	Second	First	Second	
Baseline	1135.0 ± 300.9	1082.5 ± 248.3	955.0 ± 158.9	970.0 ± 185.9	1020.0 ± 238.1	1047.5 ± 259.8	4.615*E*−4*
Signal strength	520.5 ± 77.2	502.5 ± 79.7	554.0 ± 85.5	532.2 ± 80.2	475.3 ± 66.4	470.0 ± 74.1	2.354*E*−8*
AIx@75	−0.5 ± 5.15	0.0 ± 6.07	−1.8 ± 5.52	−1.9 ± 6.54	−2.4 ± 5.71	−2.2 ± 5.90	.004*
Radial AIx	47.4 ± 10.57	47.5 ± 9.95	46.9 ± 8.12	47.6 ± 8.79	48.4 ± 9.28	47.1 ± 9.34	.552
Time to reflection	151.7 ± 9.0	151.9 ± 11.0	154.2 ± 11.0	157.9 ± 13.0	159.2 ± 13.9	158.4 ± 15.2	2.4*E*−02*
P_T2	232.4 ± 12.8	228.4 ± 31.3	231.6 ± 11.7	234.2 ± 13.1	233.3 ± 15.1	232.8 ± 12.8	.578

*The mean difference is significant at the .05 level.

**Table 3:** Comparison of the mean AIx@75 at the three palpation positions.

Position	Mean	SD	*F*	*P*-value	Duncan's test
Chon	–0.225	5.558	5.867	.004	A
Gwan	–1.825	5.974			B
Cheok	–2.275	5.729			B

**Table 4:** Comparison of the mean time to reflection at the three palpation positions.

Position	Mean	SD	*F*	*P*-value	Duncan's test
Chon	151.8	9.95	3.902	.024	A
Gwan	156.1	12.06			B
Cheok	158.8	14.36			B
